# Approaches and considerations of studying neuronal ensembles: a brief review

**DOI:** 10.3389/fncel.2023.1310724

**Published:** 2023-12-14

**Authors:** Cameron J. Davidson, Alixandria T. Mascarin, Majd A. Yahya, F. Javier Rubio, Ali Gheidi

**Affiliations:** ^1^William Beaumont School of Medicine, Oakland University, Rochester, MI, United States; ^2^Department of Psychiatry and Behavioral Neurosciences, Wayne State University School of Medicine, Detroit, MI, United States; ^3^Neuronal Ensembles in Addiction Section, Behavioral Neuroscience Research Branch, Intramural Research Program/National Institute on Drug Abuse/National Institutes of Health, Bethesda, MD, United States; ^4^Department of Biomedical Sciences, Mercer University, Macon, GA, United States

**Keywords:** cell assemblies, immediate early gene, Fos, calcium imaging, electrophysiology

## Abstract

First theorized by Hebb, neuronal ensembles have provided a framework for understanding how the mammalian brain operates, especially regarding learning and memory. Neuronal ensembles are discrete, sparsely distributed groups of neurons that become activated in response to a specific stimulus and are thought to provide an internal representation of the world. Beyond the study of region-wide or projection-wide activation, the study of ensembles offers increased specificity and resolution to identify and target specific memories or associations. Neuroscientists interested in the neurobiology of learning, memory, and motivated behavior have used electrophysiological-, calcium-, and protein-based proxies of neuronal activity in preclinical models to better understand the neurobiology of learned and motivated behaviors. Although these three approaches may be used to pursue the same general goal of studying neuronal ensembles, technical differences lead to inconsistencies in the output and interpretation of data. This mini-review highlights some of the methodologies used in electrophysiological-, calcium-, and protein-based studies of neuronal ensembles and discusses their strengths and weaknesses.

## Introduction

The idea of “cell assemblies” or “neuronal ensembles” was postulated three-quarters of a century ago in theoretical form (Hebb, 1949) and refined in the 90’s after the discovery of Fos and other immediate early genes induced by neuronal activation (Murphy et al., 1991). The study of “neuronal ensembles” has recently gained momentum in the fields of learning, memory, and motivated behavior thanks mainly to new IEGs-based, electrophysiological, and calcium imaging techniques. Neuronal ensembles represent the distributed, sparse, and distinct groups of individual neurons that are activated in response to a specific stimulus in the outside world.^1^

Our aim in this mini-review is to highlight some recent advancements in techniques for the detection and manipulation of neuronal ensembles, discuss important factors inherent to each (positive and negative), and briefly compare them. Due to constraints of the mini-review and the rapid development of this quickly growing field, some techniques or specific studies may not be included.

Early detection methods for identifying neuronal ensembles involved recording activated neurons in a physical space in the rodent hippocampus using electrophysiological recordings (Wilson and McNaughton, 1993). Wilson and McNaughton demonstrated that external space activated only particular neurons within the hippocampus, considerably influencing how we understand physiologically encoded memory. Electrophysiological recordings have demonstrated that action potentials generate calcium influx into neurons (Smetters et al., 1999). In parallel, advances in calcium sensors (Cornell-Bell et al., 1990) with sophisticated microscopy (Denk et al., 1990) allowed access to neuronal ensembles using non-electrophysiological means. This led to increased use of other methods for localizing second messengers and gene products as molecular markers correlated with neuronal electrical activity (Worley et al., 1991). In these correlational approaches, the expression of genes regulated by the cell’s electrical activity was measured in response to ongoing behavior. However, causal role experiments were not possible until neuronal ensemble-specific ablation was accomplished through genetic approaches (e.g., Daun02 chemoablation), demonstrating for the first time the co-dependence between neuronal ensembles and learned behavior (Koya et al., 2009). Fortunately, recent developments in microscopy and optogenetics have enabled researchers to study cause-and-effect relationships with non-chemogenic means, although, not all of these approaches are feasible in freely moving animal models (Adesnik and Abdeladim, 2021).

Recent experimental findings have shown that disruption of neuronal ensembles involved in behaviors related to drug abuse (Bossert et al., 2011; Fanous et al., 2012; Cruz et al., 2014; Pfarr et al., 2015; de Guglielmo et al., 2016; Caprioli et al., 2017; Xue et al., 2017; Warren et al., 2019; Gobin et al., 2022), natural reward, e.g., food (Suto et al., 2016; Quintana-Feliciano et al., 2021), social reward (Sakurai et al., 2016), and fear cues (Giannotti et al., 2019), all result in reduced corresponding related behaviors, such as reward-seeking or conditioned fear response. Electrophysiological-, calcium-, and Immediate Early Gene (IEG) -based approaches to study neuronal ensembles all present an exciting opportunity to investigate the neuronal mechanisms and connections that underlie these learned and motivated behaviors. This brief review details the strengths and weaknesses (Table 1) of the three related but different approaches to identify and manipulate neuronal ensembles.

**TABLE 1:** Representative approaches and methods for the study of neuronal ensembles.

Approach	Output measure	Detection methods	Resolution temporal/spatial	Gene/protein expression and function methods	Manipulation methods	Cause and effect?
Electrical activity-based	Action potential/single units activity	Electrophysiological recordings using tetrodes	High/limited to cells in proximity of electrodes	Electrical properties *in vivo* (Carelli et al., 1993)	Optogenetics (Taylor et al., 2014; Buzsáki et al., 2015)	Yes, if combined with optogenetics
	Action potential/fluorescence	Fluorescence voltage indicators or GEVI (e.g., ASAP type) and standard/single/two photon microscopy	High (single action potentials)/Limited to cells in proximity of the objective	Simultaneous voltage and Calcium imaging with electrical events *in vivo* (Evans et al., 2023)	Optogenetics (Fan et al., 2023)	Yes
Calcium-based	Calcium levels/fluorescence	Fluorescence Ca^2+^ indicators or GECI (e.g., GCaMP type) and single/two/multi photon microscopy	High/Limited to cells in proximity of the objective	Simultaneous Calcium imaging with electrical properties *in vivo* (Hart et al., 2022)	Optogenetics (Adesnik and Abdeladim, 2021)	Yes
		Fluorescence photoactivable Ca^2+^ integrator (e.g., CaMPARI type)	High/Poor (but permanent expression and stable *ex vivo*)	Electrical properties *ex vivo* (Trojanowski et al., 2021) FACS-based single nuclei RNA seq (O’Toole et al., 2023)	Not currently possible	Not yet (only correlational)
IEGs-based	Gene/protein expression (Fos, Arc, others)	Immunolabeling: Fos antibody *In situ* hybridization: *Arc* mRNA-based (Guzowski et al., 1999) *Fos* mRNA-based (Pfarr et al., 2018) Transgenic animals: Fos-lacZ (Koya et al., 2009) Fos-tTA (Reijmers et al., 2007) FosTRAP, ArcTRAP (Guenthner et al., 2013) FosTRAP2 (DeNardo et al., 2019)	Identifying two different time points and/or two different ensembles	Fos-based FACS couple to qPCR in wt rats (Rubio et al., 2015) Electrical properties and receptor function *ex vivo* in FosGFP mice (Koya et al., 2012) FANS-based single nucleus RNAseq (XPoSE-seq) in FosmRFP rats (Savell et al., 2023)	Fos-lacZ: Chemogenetics/beta-gal (Koya et al., 2009) Fos-tTA: Chemogenetics/DREADD (Garner et al., 2012) Optogenetic (Ramirez et al., 2013) FosTRAP2: Chemogenetics/DREADD (Koren et al., 2021) ArcTRAP: Chemogenetics/DREADD (Zuk et al., 2023)	Yes

GEVI, genetically encoded voltage indicator; GECI, genetically encoded calcium indicator; FACS, fluorescence activated cell sorting; FANS, fluorescence activated nuclear sorting; Beta-gal, β galactosidase enzyme; DREADD, designer receptor exclusively activated by designer drug; XPoSE, multipleXed population selection and enrichment.

### Electrophysiological *in vivo* recordings in freely moving rodents

Electrophysiological approaches, such as tetrode recordings, provided early evidence of neuronal ensembles and insight into relevant neurobiology by demonstrating that neuronal ensemble activation directly correlates with learned and motivated behaviors across multiple associated brain regions (Cruz et al., 2013; Kamarajan and Porjesz, 2015). Electrophysiological techniques serve to measure cellular or neuronal function in a quantitative and versatile manner (Kamarajan and Porjesz, 2015). Through electrode implantation in brain regions of interest, changes in electrical activity in the form of action potentials can be mapped to measure patterns of neuronal activity over time. Signals from multiple neurons (both excitatory and inhibitory) close in proximity to the probe comprise recordings from active brain regions in animals (Schoenbaum et al., 1999; Takahashi et al., 2003; Wikenheiser et al., 2021). Electrophysiological recordings of distinct neuronal ensembles allow for high temporal resolution in detecting ensemble activity, mapping at several depths within the brain over an extended duration of time (Buzsaki, 2004; Wenzel and Hamm, 2021). Despite the high temporal resolution, the accuracy of electrode implantation and limited recording sites restrict spatial resolution, such as when ensembles from several different brain regions are to be assessed.

### Genetically encoded voltage indicators (GEVIs)

Another promising avenue is genetically encoded voltage indicators (GEVIs). Unlike the cytosolic calcium indicators that transduce changes in intracellular calcium levels in transient fluorescence variation, the transmembrane GEVIs can sense voltage changes across the cell membrane and therefore depolarization as consequence of neuronal activity with higher temporal resolution to detect single action potentials (Liu et al., 2022; Evans et al., 2023; Fan et al., 2023). The latest improvements in GEVIs allow 2-photon deep tissue *in vivo* imaging in multiple neurons for longer times. An example of this is a jellyfish-derived electricity-reporting designer indicator for 2-photon or JEDI-2P (Liu et al., 2022). GEVIs can detect action potentials, hyperpolarization, subthreshold depolarization, and sustained depolarization (Kannan et al., 2019). Fortunately, much like calcium indicators GEVIs can be used alongside optogenetics (Fan et al., 2023).

### *In vivo* calcium imaging

Due to the direct and quantifiable association between electrical spiking and calcium influx into the neuron, calcium imaging presents an additional approach to assess cellular activation and therefore, activation of neuronal ensembles (Grienberger et al., 2022). Therefore, assessing cellular activation with real time changes in intracellular calcium levels provides an opportunity to identify activation of neuronal ensembles. This second approach to track neuronal ensembles is aided by the genetic over-expression of organic fluorescent Ca^2+^ indicators, (e.g., GCaMP; Nakai et al., 2001), and engineering advances in microscopy technology (Barbera et al., 2019; Yang et al., 2019).

Recent advancements in two-photon imaging coupled with optogenetics have seen impressive advancement in the field (Russell, 2011; Carrillo-Reid et al., 2016; Adesnik and Abdeladim, 2021; Pettit et al., 2022; Bollmann et al., 2023). Two-photon imaging can reduce image scatter endemic to traditional light and has even contributed to the development of holographic optogenetics, where sparse and distributed groups of ensembles at different brain depths are both imaged and manipulated *in vivo* (Adesnik and Abdeladim, 2021). This methodology requires a large setting, high-cost equipment, well-trained personnel, and head-fixed animals. As such, a large number of these studies have been done using sensory tasks and recording of primary sensory areas of the mouse. Thus, to perform these experiments in freely moving animals who are engaged in complex behaviors would require improvement to current designs. Fortunately, other researchers have worked on changing behavioral paradigms to fit multi-photon microscopy (Vollmer et al., 2021). This behavioral paradigm now allows *in vivo* calcium imaging using drugs as a reinforcement. A few other points of consideration are related to spatial resolution. In some of these imaging types, more superficial layers of the brain have been examined as the optical probe has penetration limits, only ensembles confined to the field of view (usually a few hundred μm can be imaged and manipulated). As the field of optical physics and microscopy advances, we are likely to see better spatial resolution to accompany the high temporal resolution that calcium imaging provides, with the ultimate goal of being conducted in freely moving animals.

More recently, the development of a calcium-modulated photoactivatable radiometric integrator (CaMPARI) has allowed identifying neuronal ensembles in free moving animals (Fosque et al., 2015; Moeyaert et al., 2018). This calcium and light dependent approach requires the use of the same tools used for optogenetic approaches but with the added advantage of being able to see the outcome *ex vivo*, in contrast to the classical GCaMP-based calcium imaging. Therefore, the CaMPARI approach allows neuronal ensembles to be detected *ex vivo* using specific antibodies to detect both states of fluorescence (active and inactive) following fluorescence photoconversion in the presence of calcium *in vivo*. Additionally, harvested tissue could be subjected to further analytical techniques such as electrophysiology, cell sorting (FACS) and RNA-seq. One advantage of this calcium-dependent photoactivation approach is the temporal specificity; the experimenter can tag the photoactive calcium sensor neuronal ensembles as soon as a few seconds after a stimulus. Additionally, CaMPARI has the potential to detect different calcium-dependent activation states by quantifying the red-to-green ratio (only red: active state, only green; inactive state) between the two fluorescent protein forms demonstrating a graduation of firing as opposed to simple binary firing indication from GCaMP sensors. A newer generation of CaMPARI, called CaMPARI2 has seen enhancements in photoconversion increasing the specificity of these graduations and allowing for a more nuanced picture of ensemble construction and activation (Trojanowski et al., 2021; Berndt et al., 2023; O’Toole et al., 2023). Researchers performing CaMPARI2 focused on activity in the cortex of head fixed mice navigating a virtual reality. Single neurons photoconverted functionally *in vivo* were sorted by fluorescence using FACS for RNA-seq analysis (O’Toole et al., 2023) demonstrating an enrichment in specific neuronal cell types within the CaMPARI2-tagged neuronal ensemble. While these calcium-based approaches are related to firing activity and offer many benefits to studying neuronal ensembles *in vivo* and *ex vivo*, they may overestimate the number of neuronal ensembles by tagging cells that, while active, are not explicitly responding to the specific stimuli (Liang et al., 2018; Tsuda, 2020; Zhou et al., 2021).

### Immediate early genes (IEGs) based approaches

As with calcium imaging and calcium-based approaches, IEGs (e.g., Fos, Arc, Zif-268) can be used as a proxy measurement for neuronal, and thus ensemble activity (Morgan et al., 1987; Guzowski et al., 1999; Guenthner et al., 2013; Beverley et al., 2014; Cruz et al., 2014; Rubio et al., 2015). IEGs have specific synaptic (Jones et al., 2001; Shepherd et al., 2006) and cellular functions (Christy et al., 1988) through their actions as transcription factors and/or effector proteins. IEG expression is rapidly induced after neuronal activation (Morgan and Curran, 1991a,b) and presents opportunities not only for identification and manipulation of ensembles, but also to study molecular alterations exclusively in neuronal ensembles (Liu et al., 2014; Li et al., 2015; Rubio et al., 2015). Traditional IEG-based techniques for neuronal ensemble detection have used IEG protein products or their nascent mRNA as indicators to identify active cells that participate in a neuronal ensemble (Kubik et al., 2007; Sauvage et al., 2019).

Additionally, given that IEGs are highly expressed in strongly activated cells, IEG-based techniques may overcome the inclusion of non-ensemble activated neurons common to both electrophysiological and calcium-based techniques. Approaches that combine the use of techniques to quantify IEG-expressing neurons and functional calcium imaging *in vivo* have shown a good correlation between IEG protein expression and neuronal (calcium) activity (Mahringer et al., 2019, 2022; Wang et al., 2021; Pettit et al., 2022). Recently, this has been conducted using 2-photon imaging to capture *in vivo* Fos ensembles (Lee et al., 2021). Though most of the brain can be visualized with this approach, the activity history of ensembles prior to the test day cannot be determined. IEG based techniques such as catFISH (Cellular compartment Analysis of Temporal activity by Fluorescent *in situ* Hybridization) based on IEGs Arc (Guzowski et al., 1999) or Fos (Pfarr et al., 2018), and Fos-based TRAP (Targeted Recombination of Activated Populations) mitigate this limitation and offer the opportunity to tag a specific ensemble at distinct time points or be able to differentiate two different ensembles in response to two distinct stimuli (Guenthner et al., 2013; Vassilev et al., 2020). The improved version, TRAP2, improves upon the first iteration of the TRAP system, including better spatial resolution as well as preservation of endogenous Fos (DeNardo et al., 2019).

Perturbation of neuronal ensembles via IEG methodology has been achieved both optogenetically (Ramirez et al., 2013) and chemogenetically (Koya et al., 2009). The Daun02 chemoablation technique allows for a timed and selective ablation of Fos-expressing ensembles, providing a powerful and causal approach to studying ensembles (Koya et al., 2009; Cruz et al., 2013). The IEG approach to identify and manipulate neuronal ensembles has been selected by different labs to demonstrate functionality and causality. The Fos-based TRAP system can be combined with the use of the Designer Receptors Exclusively Activated by Designer Drugs (DREADDs) technique. For example, a TRAP-DREADD approach has recently been applied to ameliorate neurological symptoms in a rodent model of Rett syndrome (Achilly et al., 2021). The TRAP-DREADD approach demonstrates the dual possibilities of identifying *and* manipulating these highly activated neurons in order to demonstrate the function of neuronal ensembles in encoding memories (Giannotti et al., 2019). Although using IEGs-based approaches to study ensembles offers optimal spatial resolution making them better suited to study ensembles across several brain regions or within one region at one or a few selected timepoints, the temporal resolution is not as good as can be achieved by using the calcium and electrophysiological approaches. Combining IEG-based approaches with calcium or electrophysiology techniques can solve the dissociation between spatial and temporal resolution.

## Discussion

**FIGURE 1:**
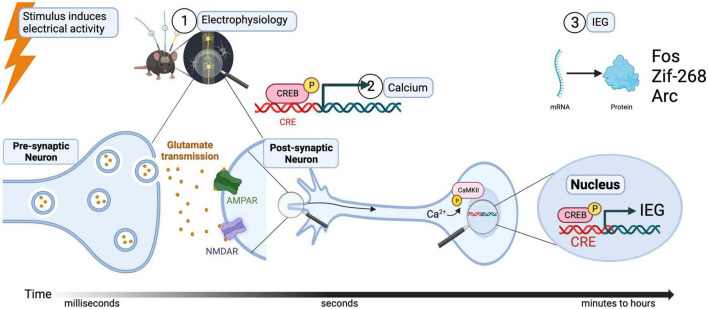
Relation of stimulus-induced neuronal activation, calcium signaling, and IEG expression.

When an action potential occurs near the cell body of a neuron ①, calcium-activated signaling can follow, either through ligand- or voltage-gated channels (Buzsaki, 2004). This influx of calcium, also known as a master switch, will ② phosphorylate Calmodulin (CaM) and in turn, Calcium/calmodulin-dependent protein kinase II (or CamKII). The destination of this second messenger cascade (starting from, for example occupation of NMDA or calcium receptors) is the cellular transcription factor cAMP response element-binding protein (or CREB) which, along with other transcription factors, ③ induces the expression of early immediate genes *Arc*, *Fos*, and *BDNF* (see Figure 1). It is important to notice that before this cascade of events occurs in the cell body of a postsynaptic neuron, information in the form of neurotransmitters released through the axon terminals of a presynaptic neuron is necessary. Therefore, signals from synapses to nuclei are also ultimately activating the soma with the consequent increase in expression of IEGs (Colgan et al., 2023). The design of new techniques and approaches to identify and modulate neuronal activation at the synaptic level is a recent area of development (Perez-Alvarez et al., 2020; Rubio et al., 2023), and although it is not the aim of this review, it is necessary to be stated as one exciting advance in the field of activated synapses.

The choice of approach or combination of techniques to study neuronal ensembles relies on the “type of activity marker” selected to identify an activated neuron (electrical activity, increase in calcium levels or IEG expression). Each activation marker represents not an undoubtedly activation state but a proxy that reveals one of the events within the activation-dependent cascade. Thus, the accuracy in identifying a neuronal ensemble is related not only to methodological aspects, feasibility, and behavioral basis of signaling to assess that question (Kim and Schnitzer, 2022), but also to the time gap between the stimulus that induces the neuronal activation and the cellular or molecular event (consequence of the activation) that the marker is revealing. The choice of methodology partly depends on whether *ex vivo* analysis will be done from the ensembles recorded *in vivo*. We may want to use electrophysiology recordings to identify functionally activated neurons in real time immediately after a stimulus, but this might overestimate the actual neuronal ensembles that are encoding a specific memory. Similarly, calcium imaging techniques detect the increase in calcium in the neuron but can’t indicate whether all neurons undergoing calcium influx are part of a specific neuronal ensemble. As the gene expression and protein translation in the nucleus is one of the ultimate events that happens after the action potential and calcium influx, and therefore needs minutes (mRNA) or hours (protein) to be detected, this methodology might reflect better the extension of a neuronal ensemble (1–5% of neurons in a region).

To overcome the limitation of individual techniques, a combination of two different approaches, such as CaMPARI as a calcium-dependent photoactivatable indicator with IEG expression can be the best way to accurately label a specific neuronal ensemble. For instance, the photoconversion of CaMPARI *in vivo* a few seconds after a specific stimulus generates a permanent change in fluorescence can be assessed *ex vivo* a few hours later in combination with Fos immunohistochemistry. Analyzing the neurons that express both the active CaMPARI form and Fos protein might give us better resolution to identify specific neuronal ensembles. This method will control for neuronal ensembles that are actually composed of mixed populations of neurons activated by specific stimuli and those triggered by cues, contexts which are also picked up by classical electrophysiology or calcium imaging methods. Some of these combined approaches have recently started, the combination of 2-photon imaging the contribution of GABAergic vs. Glutamatergic neurons in shaping ensembles in the barrel cortex (Bollmann et al., 2023; Kourdougli et al., 2023), two-photon imaging has also established a cause -and-effect link between Fos and place cell activity (Pettit et al., 2022), Fos positive neurons are a more reliable index of place places than non-positive Fos cells. A surprising twist to the story has also come from the combined use of *in vivo* 2-photon and Fos imaging (Lee et al., 2021). The authors show that whisker-dependent sensory association learning in the primary somatosensory cortex does not alter Fos, and in fact synaptic changes are predominant in non-Fos cells. As referenced earlier in this paper, the approaches are not necessarily competing; but rather, each may be used to answer unique questions about how neuronal ensembles contribute to the neurobiology of motivated behavior. Future investigations, especially those that are purporting to study “neuronal ensembles” need to agree upon a common, operational definition of what this entails and distinction from engrams as we have included. Integration and exploration of these approaches, guided by their prior use in other domains, can provide great insights into future research on “neuronal ensembles/cell assemblies.”

## Author contributions

CD: Conceptualization, Writing – original draft, Writing – review and editing. AM: Conceptualization, Software, Writing – original draft, Writing – review and editing. MY: Writing – original draft, Writing – review and editing. FJR: Formal analysis, Supervision, Writing – original draft, Writing – review and editing. AG: Funding acquisition, Supervision, Writing – original draft, Writing – review and editing.

## Footnotes

1 We would like to make a clear distinction between *Neuronal Ensembles* and *Engrams*. We are defining ensembles using the theoretical definition from Hebb (1949) as a sparse distributed pattern of cells/synapses that are selectively activated by cues or stimuli to mediate specific information, while engrams are defined as long-lasting molecular and/or cellular adaptations that can physically encode learned associations within these ensembles.
